# Submergence Tolerance and Germination Dynamics of *Roegneria nutans* Seeds in Water-Level Fluctuation Zones with Different Water Rhythms in the Three Gorges Reservoir

**DOI:** 10.1371/journal.pone.0151318

**Published:** 2016-03-31

**Authors:** Feng Lin, Jianhui Liu, Bo Zeng, Xiaojiao Pan, Xiaolei Su

**Affiliations:** Key Laboratory of Eco-environments in the Three Gorges Reservoir Region (Ministry of Education), Chongqing Key Laboratory of Plant Ecology and Resources Research in the Three Gorges Reservoir Region, School of Life Sciences, Southwest University, Chongqing, 400715, PR China. These authors contributed equally to this work; Shandong University, CHINA

## Abstract

The Three Gorges Dam features two water-level fluctuation zones (WLFZs): the preupland drawdown zone (PU-DZ) and the preriparian drawdown zone (PR-DZ). To investigate the vegetation potential of *Roegneria nutans* in WLFZs, we compared the submergence tolerance and germination dynamics in the natural riparian zone (NRZ), PU-DZ and PR-DZ. We found that the NRZ seeds maintained an 81.3% intactness rate and >91% germination rate. The final seed germination rate and germination dynamics were consistent with those of the controls. Meanwhile, the PU-DZ seeds submerged at 5 m, 10 m, 15 m, and 20 m exhibited intactness rates of 70.5%, 79.95%, 40.75%, and 39.87%, respectively, and >75% germination. Furthermore, the PR-DZ seeds exhibited intactness rates of 22.44%, 61.13%, 81.87%, and 15.36% at 5 m, 10 m, 15 m, and 17 m, respectively, and 80% germination. The germination rates of the intact seeds submerged >10 m were >80%. Finally, the intact seeds germinated quickly in all WLFZs. The high proportion of intact seeds, rapid germination capacity, and high germination rate permit *R*. *nutans* seeds to adapt to the complicated water rhythms of the PU-DZ and PR-DZ and indicate the potential for their use in vegetation restoration and recovery. Thus, perennial seeds can be used for vegetation restoration in the WLFZs of large reservoirs and in other regions with water rhythms similar to the Three Gorges Reservoir.

## Introduction

The construction of large-scale reservoirs benefits humanity in terms of flood prevention, navigation and power generation. However, these reservoirs are associated with multiple ecological and environmental issues, such as vegetation degradation and severe water and soil loss in the bank drawdown zone [1,2]. As a riparian zone of the reservoir bank ecosystem, the drawdown zone is a link for matter and energy exchange for the aquatic-terrestrial ecosystem. Moreover, as a basis for the ecosystem function of the drawdown zone, vegetation plays a crucial role in withstanding erosion and preserving water and soil in this zone [3,4]. However, the terrestrial vegetation existing in the area before the reservoir construction is degraded by the complicated hydrological conditions formed after its construction [5–7]. After reservoir filling, the degree of vegetation coverage and species richness decrease significantly in the riparian zone; trees, shrubs, and perennial herbs are only distributed zonally in high-elevation regions in the riparian zone, and only a small number of perennial and annual herbs grow in low-elevation regions, where water flooding intensity is strong [8,9]. Vegetation restoration and recovery in the reservoir riparian zone is of great significance in maintaining the stability of the reservoir bank zone to safeguard the ecosystem function and the functional integrity of the drawdown zone [10,11].

Plants adaptable to the environment of the reservoir riparian zone should be chosen for vegetation restoration of the reservoir riparian zone [2]. At present, the selection of plants growing in the reservoir riparian zone focuses on two approaches. The first approach entails selecting perennial plants with strong resistance to flooding and planting their seedlings or tillering directly in water-level fluctuation zones (WLFZs) for vegetation restoration [12–14]. The second approach is the selection of annuals whose seeds can survive the flooding period to grow and multiply during the exposed period in WLFZs; the seeds of these annuals are sown in these zones for vegetation restoration [15,16]. The first approach is effective but requires considerable manpower and material resources. The second method is less effective than the first but is economical and practical due to the increase in vegetation cover resulting from seed distribution. Perennials whose plants and seeds can tolerate different intensities of water submergence and whose seeds can germinate rapidly after water recession would allow the benefits of both methods to be exploited simultaneously. The availability of such perennials for revegetation in the riparian zone would not only guarantee effective restoration but also conserve manpower and material resources.

Seeds used for vegetation restoration in WLFZs must tolerate flooding in the reservoir riparian zone and germinate quickly into seedlings for colonization during the exposed period. Large reservoirs exhibit various hydrological rhythms and tremendous water-level fluctuations, and the characteristics of the flooding seasons, including duration, submergence depth and frequency, are key factors influencing planting in WLFZs [17,18]. In wetlands in which vegetation has been constructed using soil seed banks, species richness and the degree of coverage of the plant community decrease with increasing water submergence depth, duration and frequency after water recession. Among these three factors, the submergence depth has the smallest influence, whereas the submergence duration has the greatest influence [19]. In addition, the season in which submergence occurs noticeably influences the composition of the soil seed bank. Holzel and Otte [20] investigated the influence of water submergence in summer on the species richness and degree of coverage along the Upper Rhine River and observed that the intensity of the seeds of summer annuals and perennials that require high germination temperatures significantly decreased, whereas winter annuals and perennials that require low germination temperatures did not differ significantly, possibly due to differences in water submergence tolerance among seeds of different species due to differences in environmental requirements for breaking dormancy and germination. *Cyperus squarrosus* has a satisfactory water submergence capacity in varying hydrological environments but can only germinate well at midsummer temperature (35°C/20°C) [21]. *Hottonia inflata* exhibits good summer water submergence tolerance but does not germinate until autumn and winter [22]. The environment the seeds are exposed to in the riparian zone after water submergence directly influences their germination capacity. In the riparian zones of large reservoirs, the characteristics of the hydrological rhythms, such as water submergence season, duration and frequency, are closely associated with the geographical locations of the riparian zones. The hydrological rhythms and the environment that plants are exposed to after water recession in the riparian zones may vary greatly among different geographical locations, which inevitably leads to differences in the water submergence tolerance and germination capacities of the seeds in the riparian zones at different locations. Therefore, studying and verifying the seed submergence tolerance and germination capacity of a plant are of practical value for vegetation restoration under multiple hydrological rhythms in the reservoir riparian zone.

**Fig 1:**
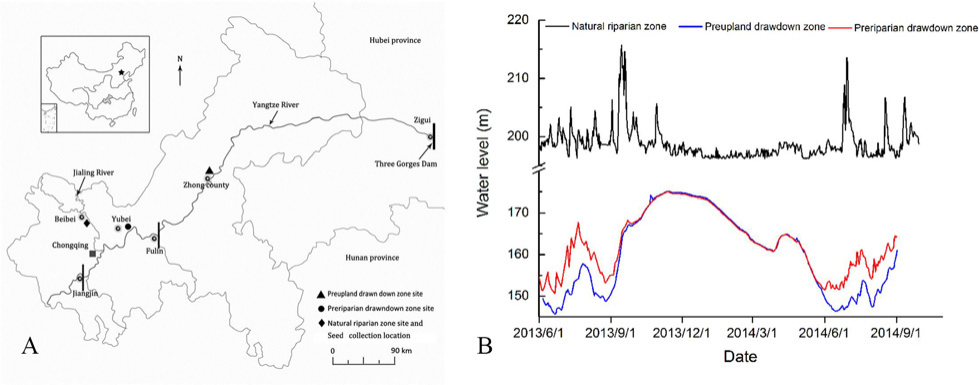
Location of the Three Gorges Reservoir, experiment sites and water levels for the three types of drawdown zones used in the experiment. A, experiment sites. B, water levels in the experiment.

The Three Gorges Reservoir is one of the largest reservoirs in the world, with a reservoir bank zone of nearly 660 km, a drawdown zone of approximately 380 km^2^, and a maximum vertical drop of 30 m in the WLFZ (Fig 1A). After its completion in 2008, the following two types of drawdown zones with different water level rhythms were formed in the bank zone: the preupland drawdown zone (PU-DZ), which is largely influenced by the reservoir winter impoundment from Fuling to the dam section, and the preriparian drawdown zone (PR-DZ), which is affected by both summer flooding and winter reservoir impoundment, starting from Jiangjin to Fuling [7,23]. The PR-DZ is submerged from September to May due to the reservoir winter-spring impoundment, flooded in July and August, and quickly submerged again by reservoir impoundment after the flood recedes (Fig 1B). Compared with the natural riparian zone (NRZ, only influenced by the natural water regime of the Yangtze River), fewer species were found per 100 m^2^ in the PR-DZ than in the NRZ, and forbs, ferns and graminoids decreased significantly [7]. The vegetation stability and water and soil conservation capacity in the area are severely damaged and may be even worse than those in the PU-DZ [24]. Vegetation restoration and recovery are the main measures used to enhance ecosystem stability and reduce water and soil erosion in the reservoir drawdown zone [25]. However, the literature has mainly concentrated on vegetation restoration and species selection for the PU-DZ in the Three Gorges Reservoir; few studies have addressed species selection for the PR-DZ based on its hydrological conditions.

*Roegneria nutans* is a variety of Gramineae Roegneria that is widely distributed in subtropical and temperate regions. *R*. *nutans* grows on slopes and in wet grassland at elevations ranging from 100 m to 2300 m throughout China, except in Tibet and Qinghai. *R*. *nutans* is common along natural river banks and can be found in the WLFZs of the Three Gorges Reservoir [26]. *R*. *nutans* has strong tillering and regeneration capacities, turns green every spring (March to May), and bears many mature seeds from April to July. Its tillering node features dense root heads and therefore has good capacities for water and soil conservation. This experiment examined the survival and germination capacities of *R*. *nutans* subjected to a complete natural hydrological process in NRZ, PU-DZ and PR-DZ and explored the potential use of its seeds in vegetation recovery during the exposed period in the reservoir drawdown zone, especially in the PR-DZ. Our findings provide a reference for the selection of perennial seeds for vegetation restoration in large reservoirs, in addition to alternative species for vegetation restoration in the various WLFZs of large reservoirs similar to the Three Gorges Reservoir.

## Materials and Methods

### Experiment sites

The Three Gorges Reservoir (106°-111°50′E, 29°16′-31°25′N, Fig 1A) has a subtropical monsoon climate with an annual average temperature of 15–19°C, an average rainfall of approximately 1,250 mm, and a relative humidity of 76% [27]. In the experiment, the main pier of the Beibei section of the Jialing River, which is an important tributary of the Yangtze River located in the tail area of the Three Gorges Reservoir, was selected as the flooding experiment site for the NRZ. Ganxizi, in Luoqi Township, Yubei District, and the middle part of the PR-DZ of the Three Gorges Reservoir (the section stretching from the Jiangjin section to the Fuling section of the Yangtze River), was selected as the flooding experiment site for the PR-DZ. The vegetation restoration demonstration zone for the Three Gorges Reservoir riparian zone, located in Tujing Township, Zhongxian County in the hinterland of the Three Gorges Reservoir, was selected as the flooding experiment site for the PU-DZ. To conveniently observe seed germination after the various flooding experiments, the germination experiment for examining all seeds after flooding was conducted on the Jialing River bank soil outdoor germination bed within the Ecological Park of Southwest University, in Beibei District, Chongqing Municipality. As Southwest University is located in the Three Gorges Reservoir, the natural conditions (e.g., temperature and sunlight) of which are similar to those of the reservoir bank zone, the germination bed sufficiently simulated the natural germination conditions of the plant seeds growing in the reservoir bank zone. The location of the Three Gorges Reservoir, experiment sites and water levels for the three types of drawdown zones studied are as indicated in Fig 1.

This study was approved by the Department of Technology and Environmental Protection of the Three Gorges Group, China.

### Methods

#### Collection and counting of seeds

During the maturation period of *R*. *nutans* seeds, seeds were gathered from multiple plants along the bank of the Jialing River (106°26′48′′E, 29°49′09.6′′N) and air-dried indoors. Seeds of full grain and uniform size were selected and counted. Each sample consisted of 200 grains placed in a tied nylon seed bag to prevent seed loss.

#### Unflooded control group

The control group seed bags (five bags with 1,000 seeds in each) were closely sown over the soil surface of the germination bed within the Ecological Park of Southwest University without being flooded. Seed germination and dead seedlings were observed monthly, and the seedlings were removed. Radicle breaking through the seed coat to a length of 2 mm was considered germination for the mature *R*. *nutans* seeds under natural conditions.

#### Summer flooding group

To ensure for uniform flooding intensity throughout the flooding season, *R*. *nutans* seed bags were fixed on a barge on the main pier of the Jialing River to maintain a submergence depth of 2 m during the flooding season. Five samples (bags) were established for the summer flooding experiments; each nylon sample bag contained 200 seeds. The concrete flooding times are presented in Table 1. After flooding, we quickly recorded whether the remaining seeds in each bag were in good condition. If the seed structure was complete and solid, or if it was incomplete but the embryo structure of the seed was complete and the endosperm was white, the seed was considered to be intact [28]. The intact seeds were then returned to their original bags. Subsequently, these seeds were closely sown into the soil surface of the germination bed, which consisted of soil taken from the bank zone, and were covered with a 2-mm layer of sandy soil. Seed germination and dead seedlings were recorded monthly to compare seed germination with that in the unflooded control group, and the seedlings were removed.

**Table 1:** Details of the submergence experiments for the three types of WLFZs.

Type of WLFZ	Flooding season	Submergence depth (above sea level)	Flooding starting time	Flooding ending time
**Unflooded control group**	None	None	None	None
**NRZ**				
	Natural flooding	2 m	2013.7.15	2013.9.15
**PR-DZ**				
	Flooding in summer	2 m	2013.7.15	2013.9.15
	Reservoir impoundment flooding in winter			
		5 m (170 m)	2013.10.13	2014.1.21
		10 m (165 m)	2013.9.15	2014.2.18
		15 m (160 m)	2013.9.11	2014.3.26
		17 m (158 m)	2013.9.6	2014.5.11
**PU-DZ**				
	Reservoir impoundment flooding			
		5 m (170 m)	2013.10.14	2014.1.21
		10 m (165 m)	2013.9.17	2014.2.19
		15 m (160 m)	2013.9.12	2014.5.5
		20 m (155 m)	2013.9.8	2014.5.15

#### Double flooding by summer flooding and winter impoundment

(1) *Summer flooding processing* To ensure consistency between the processing methods in the summer flooding portion of the flooding-impoundment experiment and in the natural flooding experiment, 20 bags of *R*. *nutans* seeds were divided into 4 groups of 5 seed bags each, and the 4 groups were submerged in the PR-DZ at 5-m, 10-m, 15-m, and 17-m water depths in the winter impoundment after the same summer flooding. After the bags were marked, they were fixed onto a barge on Beibei’s main pier on the Jialing River. During the flooding season, all seed bags were maintained at a submergence depth of 2 m, and the flooding duration was approximately 60 days (Table 1). After the flooding ended, the number of seeds that remained intact was quickly recorded, and the intact seeds were returned to their original bags.

Because the flooding caused by the reservoir impoundment varied by elevation in the WLFZs, to simulate the natural living conditions of seeds in the field at different elevations, the bags in each group were closely sown over the soil surface on a germination bed composed of soil from the river bank after the seed intactness was recorded. When the corresponding elevations in the PR-DZ were to be flooded by the reservoir impoundment, seed germination was observed and recorded to compare seed germination with that in the unflooded control group and the seedlings were removed.

(2) *Processing for winter impoundment after summer flooding* Elevations of 158 m, 160 m, 165 m and 170 m (corresponding to submergence depths of 17 m, 15 m, 10 m, and 5 m, respectively) in the riparian zone in Luoqi township, Yubei District, located in the PR-DZ were flooded by the impoundment in the drawdown zone. Prior to this flooding, a group of seed bags on the germination bed was selected for each elevation, and the numbers of intact seeds remaining and of germinated seeds were recorded. The intact seeds were returned to their original bags, which were placed at the abovementioned elevations. Therefore, for each elevation, there were 5 replicate samples. The seed number in each repeated sample was the seed number in the bags for the winter impoundment flooding experiment. Immediately before each elevation was exposed, the seed bags at that elevation were recovered (Table 1). To ensure that the seeds did not influence the follow-up experiment due to water shortage during transportation in the recovery process, the seed bags were kept in a flooded environment. After the bags were recovered, the number of intact seeds remaining was quickly recorded, and the intact seeds were returned to their original bags and placed on the germination bed in the Ecological Park of Southwest University. Seed germination and dead seedlings were recorded monthly for comparison with the unflooded control group, and seedlings were removed.

#### Flooding by winter impoundment

Four flooded gradients were set up in the vegetation restoration demonstration zone in the reservoir riparian zone in Zhongxian County at 155 m, 160 m, 165 m and 170 m (with submergence depths of 20 m, 15 m, 10 m, and 5 m, respectively) (See Table 1). Each elevation (submergence depth) had 5 repeated samples of 200 seeds per sample. Before the corresponding elevation was flooded, the seed bags were fixated at the corresponding elevation for the winter impoundment flooding, and the flooding and post-flooding processing were the same as those for the winter impoundment portion of the dual flooding experiment.

#### Data processing and plotting

After flooding, the proportion of seeds remaining intact was calculated as L = S/T×100%, where L is the proportion of the intact seeds after flooding, S is the number of remaining intact seeds after flooding, and T is the number of total seeds for the flooding experiment. The proportion of the remaining intact seeds represents the submergence tolerance of the seeds.

The cumulative germination rate was calculated as *G* = *G*i/*R*×100%, where G is the germination rate until time *i*, *Gi* is the total number of germinated seeds until time *i*, and *R* is the total number of seeds used for the germination experiment.

The final seed germination rate can be represented as *P* = *gt*/*T*×100%, where *gt* is the total number of seedlings germinated from the remaining seeds in the sample under natural conditions after flooding in the different WLFZs and *T* is the total number of seeds used for the flooding experiment. The final seed germination rate (*P*) represents the maximum seedling potential of the total seeds submerged after flooding in the different WLFZs.

Excel 2010 (Microsoft Inc.) was used for the data reduction. One-way ANOVA (DUNCAN) in SPSS 20.0 was employed to analyze the significance of the intact seed rate for flooded seeds at different elevations within the same WLFZ and the differences among germination rates. The independent sample *t*-test was adopted for significance analysis of the final seed germination rates for the different flooded elevations for the different flooding types. Origin 9.1 (Origin, Inc.) was used to generate diagrams.

## Results

### Submergence tolerance of *R*. *nutan*s seeds for the three types of WLFZs with different water rhythms

*R*. *nutans* seeds exhibited good submergence tolerance for summer flooding (Table 2). After two months of summer flooding, the seeds exhibited an intactness rate of >81%. Although the seeds experienced some decay after summer flooding, the germination rate of the remaining seeds under natural conditions after the flooding was significantly higher than that of the natural field control group, which did not experience summer flooding (p<0.05), and the final seed germination rate did not differ from that of the unflooded control group (p>0.05).

**Table 2:** Submergence tolerance of *R*. *nutans* seeds after summer flooding in the riparian zone of Jialing river (mean±SE).

Flooding type	Submergence depth (m)	Intactness rate (%)	Germination rate of intact seeds (%)	Final seed germination rate (%)
**Unflooded control group**	0	——	74.7±2.24 a	74.7±2.24 A
**Summer flooding in natural rivers**	2	81.3±2.3	91.41±3.46 b	74.3±0.94 A
**Summer flooding phase for the PR-DZ**	2	81.25±1.15	——	——

The lowercase letters a and b indicate significant differences between the germination rates of the natural field control seeds and the remaining intact seeds subjected to natural summer flooding (p<0.05). The uppercase letter A indicates the lack of a significant difference between the final seed germination rate of *R*. *nutans* seeds subjected to natural summer flooding and that of the unflooded control seeds, which did not experience summer flooding. An em-dash indicates that the parameter was not determined in this study.

In the PU-DZ, *R*. *nutans* seeds were only subjected to flooding by winter impoundment, and the proportion of intact seeds tended to decrease with increasing submergence depth. However, even when the submergence depth reached 20 m and the flooding duration was approximately 8 months, the intactness rate was approximately 40%, and their germination rate was >75% (Table 3).

**Table 3:** Submergence tolerance of *R*. *nutans* seeds within the winter impoundment of the Three Gorges Reservoir (mean±SE).

Submergence depth (m)	Flooded by winter impoundment in the PR-DZ		Flooded by winter impoundment in the PU-DZ	
	Proportion of intact seeds	Cumulative germination rate	Rate of intact seeds	Cumulative germination rate
**5**	24.44±9.55 a	53.34±13.68 A	85.33±1.48 i	90.38±4.05 I
**10**	64.13±8.99 b	87.52±3.05 B	79.95±3.64 i	86.75±7.14 I
**15**	81.87±4.4 c	95.37±0.8 C	40.45±5.48 ii	79.63±8.25 II
**17**	15.36±3.05 a	83.88±5.54 B	——	——
**20**	——	——	39.87±6.49 ii	76.06±2.15 II

The lowercase letters a, b, c, d, i and ii indicate significant differences between the proportions of remaining intact *R*. *nutans* seeds subjected to winter impoundment in the PR-DZ and those subjected to full flooding by winter impoundment in the PU-DZ at different elevations. The uppercase letters A, B, C, D, I and II indicate significant differences between the cumulative germination rates of the remaining intact seeds under natural conditions (p<0.05). An em-dash indicates that the parameter was not determined in this research.

After flooding in the PR-DZ, the intactness of the *R*. *nutans* seeds at different submergence depths exhibited a tendency toward rising and then decreasing. After 2 months of summer flooding and approximately six months of winter impoundment at a submergence depth of approximately 15 m, the seeds maintained an 81.87% intactness rate and a 95.37% germination rate. Although the submergence depth in the PR-DZ was 17 m and the flooding duration was approximately 8 months, the intactness rate of the seeds was only 15.36%. However, the seeds exhibited a high germination rate of 83.88% (Table 3).

**Fig 2:**
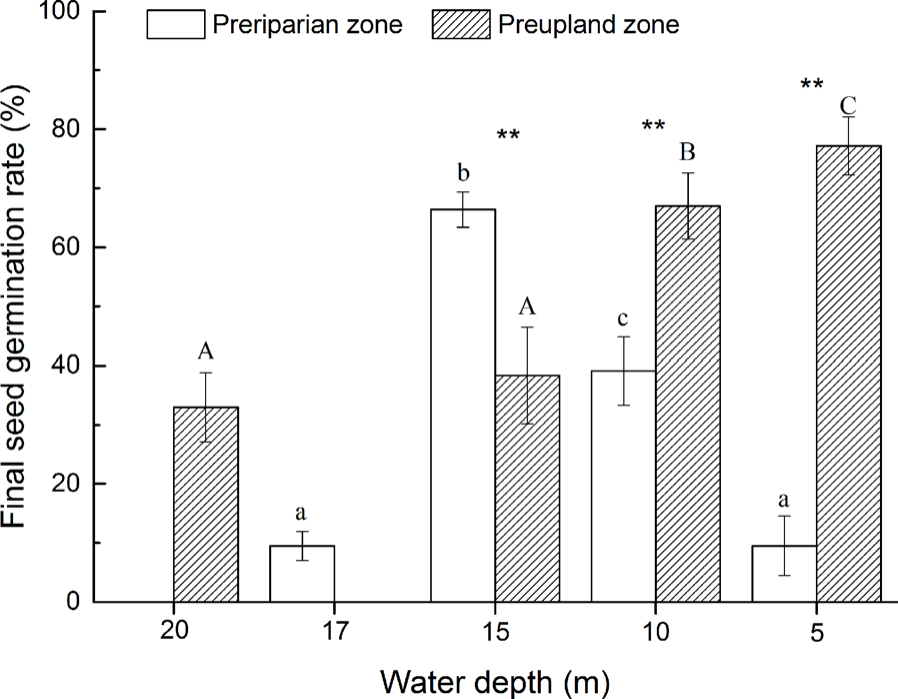
Final seed germination rate of *R*. *nutans* seeds submerged at different elevations in the PR-DZ and PU-DZ. The lowercase letters represent the differences between the final seed germination rates at different elevations in the PR-DZ, whereas the uppercase letters represent the differences between the final seed germination rates at different elevations in the PU-DZ. Bars with the same letter are not significantly different, whereas those with different letters are significantly different, p<0.05. ** Represents significant differences between the final seed germination rates at the same elevation between different WLFZs, p<0.05.

After being subjected to flooding by winter impoundment following summer flooding, the final potential seedling rate of *R*. *nutans* seeds exhibited a tendency toward rising and then declining with increasing submergence depth. When the submergence depth was 15 m (at an elevation of 160 m), the seedling rate reached its maximum of 66.4%. For *R*. *nutans* seeds directly subjected to winter impoundment, the seedling rate exhibited a declining tendency with increasing submergence depth in the WLFZ, and when the submergence depth reached 20 m (at an elevation of 155 m), the seeds exhibited a 33% final germination rate (Fig 2).

### Germination dynamics of *R*. *nutans* seeds after being submerged in WLFZs with different water rhythms

**Fig 3:**
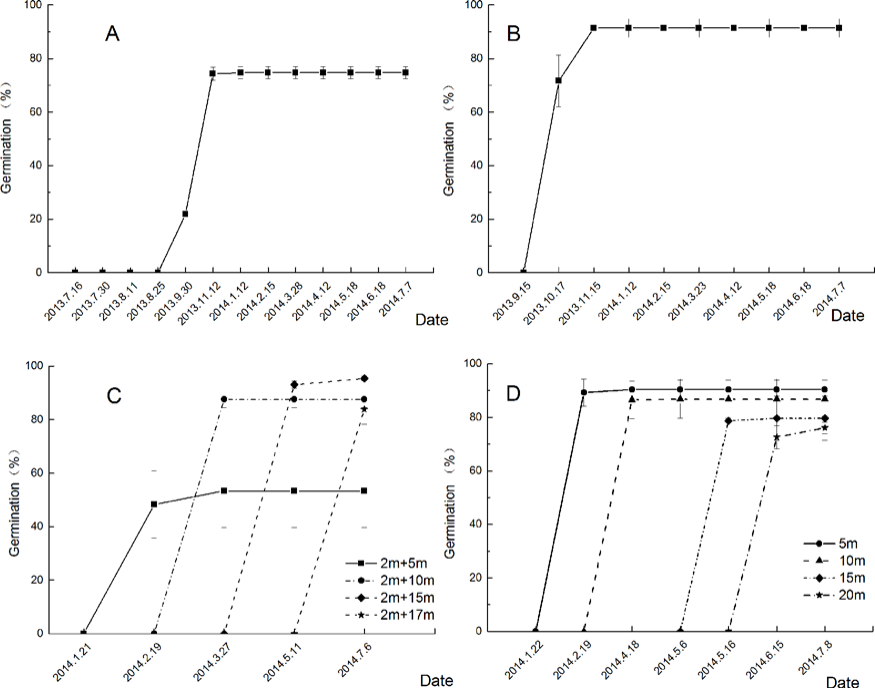
Germination dynamics of *R*. *nutans* seeds after being submerged in WLFZs with different water rhythms. A refers to the germination dynamics of *R*. *nutans* seeds that were not submerged under natural conditions. B, C, and D refer to the germination dynamics of the remaining seeds submerged by natural summer flooding (with a submergence depth of 2 m and a flooding duration of 2 months), by summer flooding (with a submergence depth of 2 m and a flooding duration of 2 months) followed by flooding by winter impoundment, and by flooding by winter impoundment at different submergence depths, respectively.

Natural summer flooding did not change the germination dynamics of *R*. *nutans* seeds. After being subjected to natural summer flooding, the seeds exhibited the same germination dynamics as those that were not subjected to summer flooding. Furthermore, flooding increased the germination rate of *R*. *nutans* seeds (Fig 3A and 3B).

Anti-seasonal flooding in the Three Gorges Reservoir riparian zone delayed the germination period of *R*. *nutans* seeds until spring and summer of the following year. Nonetheless, *R*. *nutans* was still able to quickly realize massive seed germination (Fig 3A, 3B, 3C and 3D). *R*. *nutans* seeds in the PU-DZ and PR-DZ subjected to natural conditions after exposure to water could no longer germinate. However, seeds at different elevations exhibited >76% germination within one month after exposure to water in the PU-DZ (Fig 3D). By contrast, seeds treated at >10 m submergence depth in the PR-DZ exhibited >83% germination within one month after exposure to water. Although the germination rate was low at a submergence depth of 5 m, the seeds still exhibited >53% germination (Fig 3C).

## Discussion

Vegetation restoration for large reservoirs is an important means of water and soil conservation, pollution cleanup in WLFZs, and maintenance of the system structure and functional completeness of reservoir bank ecology. Using perennial seeds for vegetation restoration in WLFZs is highly cost-effective. However, seeds’ tolerances to submersion in different types of WLFZs of large reservoirs and their germination capacities during the exposed period determine their true capacities for vegetation restoration in these zones. As the Three Gorges Reservoir is one of the largest reservoirs in the world, much of the original vegetation in the WLFZs has died out due to the deep, long-term, and anti-seasonal flooding. Vegetation restoration is a long-term task. The ability of seeds of *R*. *nutans*, a perennial found in the bank of the Yangtze River, to withstand submergence under different hydrological rhythms in the Three Gorges Reservoir and to rapidly germinate and grow in the reservoir’s different WLFZs is an important determinant of its vegetation restoration capacity at this location.

*R*. *nutans* seeds subjected to summer flooding in the NRZ exhibited a high intactness rate of approximately 81% (Table 1). Furthermore, summer flooding facilitated the germination of the remaining intact seeds, which exhibited a germination rate of 91% (Table 2 and Fig 3A and 3B). This high germination rate is important for the plant’s ability to spread and maintain its population in the river bank, demonstrating its adaptability to natural flooding as a common plant in the river bank [29]. When *R*. *nutans* seeds were directly subjected to winter impoundment, the proportion of remaining intact seeds, the germination rate, and the final seed germination rate all exhibited a declining tendency with increasing submergence depth (Table 3 and Fig 2). However, when the seeds were subjected to natural summer flooding and then flooding by winter impoundment, the proportion of the remaining intact seeds and germination rate both exhibited a rising and then declining tendency (Table 3 and Fig 2), which might have been because flooding was delayed at the higher elevation (170 m), and its occurrence coincided with germination under natural conditions. Seeds at this elevation were not submerged by impoundment after experiencing summer flooding, and the germination period of *R*. *nutans* seeds occurs in the middle of October under natural conditions. When *R*. *nutans* seeds were placed at elevations that experience winter impoundment (Table 1), the seeds germinated underwater and then rose above the waterline and decayed [30,31]. Moreover, during the high water level periods of the winter impoundment of the Three Gorges Reservoir, the water level fluctuates drastically between elevations of 170 m and 175 m, contributing to the increase in the seed rotting rate at 170 m.

Under natural conditions, the seeds of *R*. *nutans* that were not subjected to water submergence (the control) had a final germination rate of 74.7%, and germination primarily occurred between September and October. The seeds of *R*. *nutans* that underwent natural flooding had a final germination rate of 74.3%, and their germination time was nearly identical to that of the control. Winter impoundment of the Three Gorges caused a delay in the germination time of the seeds in the PU-DZ and PR-DZ at different elevations due to water recession, and the germination dynamics were similar at the same elevations in the PU-DZ and PR-DZ (Fig 3C and 3D), the final germination rates of the PU-DZ at 5 m and 10 m were both approximately 70%, nearly identical to those of the control and NRZ groups but significantly higher than that of the PR-DZ group. The germination rate of the PU-DZ 20-m treatment group was higher than that of the 17-m PR-DZ group, but the final germination rates of the PU-DZ and PR-DZ groups with water submergence depths > 15 m were both 40% (Fig 2). These results indicate that deep water submergence decreased the germination of *R*. *nutans* seeds and that the double-submergence of summer flooding and winter impoundment had a greater influence. The primary factor in the decrease in the final germination rates of the PU-DZ and PR-DZ groups was the decrease in the intactness rate of the seeds; the germination rates of seeds remaining intact in both groups were greater than 76% except for the 5-m PR-DZ group, for which the germination rate was 53.34% (Table 3). These results demonstrate that water submergence promoted the germination of *R*. *nutans* seeds to some degree, as indicated not only by the increase in the germination rate of the intact seeds above the water level but also by the acceleration of germination progress by 1–2 months (Fig 3). This enhanced germination may be due to breaking of dormancy due to water submergence and an improved environment for germination after water recession [21,22].

After the *R*. *nutans* seeds were subjected to submergence in the three types of WLFZs and exposed to water, 50% to 80% of the remaining intact seeds germinated within a month (Fig 3). This germination behavior demonstrates the species’ extensive adaptability under different environments [32,33]. For water and soil conservation and the stability of the ecological system in the WLFZ, rapid germination behavior is conducive to vegetation restoration in the exposed drawdown zone after exposure to water because it can strengthen the retention of water and soil in this zone [34], increasing the species diversity of the ecological system and its stability [35]. *R*. *nutans* seeds exhibited good submergence tolerance and germination characteristics for adaptation to the river bank environment. These seeds germinate rapidly after being subjected to summer flooding and flooding by winter impoundment, which guarantees a longer growth period after germination prior to the next flooding and establishes a competitive advantage through massive germination.

This study investigated the vegetation restoration capacity of *R*. *nutans* seeds in different types of WLFZs in the Three Gorges Reservoir only from the perspective of their submergence tolerance in different types of WLFZs and their germination capacity after being submerged. We did not examine the plant’s submergence tolerance or its ecological function in WLFZs with different water rhythms. The experimental results indicate that *R*. *nutans* seeds exhibit very good adaptability in the different types of WLFZs of the Three Gorges Reservoir. However, we did not examine the physiological or molecular mechanisms of the adaptation process. Further research should focus on the effects of field restoration and elucidate how *R*. *nutans* can adapt to the long and anti-seasonal deep flooding in the WLFZs of the Three Gorges Reservoir from physiological, biochemical and molecular perspectives. In addition, the duration of submergence in the 15-m treatment differed between the PR-DZ and PU-DZ groups due to water-level fluctuations during the experiment, which might have led to a higher intactness rate in the PR-DZ 15-m treatment group than would normally occur under these conditions. However, even if the seeds were out of the water for identical lengths of time in the 15-m PR-DZ and PU-DZ groups, the intactness rate of the 15-m PR-DZ group should be higher than that of the 17-m PR-DZ group, and its germination rate would be higher than 75%. Therefore, the seeds at the 15-m elevation of the PR-DZ still had satisfactory application potential.

By maintaining a high proportion of intact seeds during the flooding process and a rapid germination capacity after exposure to water, *R*. *nutans* seeds adapt well to complicated and long-term hydrological rhythms that do not occur in the plant’s NRZ. *R*. *nutans* exhibits great potential for application in vegetation reconstruction and restoration during the exposed period, especially in the PR-DZ. Our research also demonstrates the practicality of using perennial seeds for vegetation restoration in the WLFZs of large reservoirs. The results of this study can also be used to promote vegetation recovery and restoration in other regions with similar water rhythms as the Three Gorges Reservoir.
